# Electrochemical DNA Hybridization Sensors Based on Conducting Polymers

**DOI:** 10.3390/s150203801

**Published:** 2015-02-05

**Authors:** Md. Mahbubur Rahman, Xiao-Bo Li, Nasrin Siraj Lopa, Sang Jung Ahn, Jae-Joon Lee

**Affiliations:** 1 Nanotechnology Research Center and Department of Applied Life Science, College of Biomedical and Health Science, Konkuk University, Chungju 380-701, Korea; E-Mails: mahbub1982@kku.ac.kr (M.M.R.); xiaobo@kku.ac.kr (X.-B.L.); lopa@kku.ac.kr (N.S.L.); 2 Center for Advanced Instrumentation, Korea Research Institute of Standards and Science (KRISS), Daejeon 305-340, Korea; E-Mail: sjahn@kriss.re.kr

**Keywords:** electrochemical DNA sensors, conducting polymers, immobilization techniques, transduction mechanism

## Abstract

Conducting polymers (CPs) are a group of polymeric materials that have attracted considerable attention because of their unique electronic, chemical, and biochemical properties. This is reflected in their use in a wide range of potential applications, including light-emitting diodes, anti-static coating, electrochromic materials, solar cells, chemical sensors, biosensors, and drug-release systems. Electrochemical DNA sensors based on CPs can be used in numerous areas related to human health. This review summarizes the recent progress made in the development and use of CP-based electrochemical DNA hybridization sensors. We discuss the distinct properties of CPs with respect to their use in the immobilization of probe DNA on electrode surfaces, and we describe the immobilization techniques used for developing DNA hybridization sensors together with the various transduction methods employed. In the concluding part of this review, we present some of the challenges faced in the use of CP-based DNA hybridization sensors, as well as a future perspective.

## 1. Introduction

Deoxyribonucleic acid (DNA) detection plays a prominent role in myriad disciplines related to human health, including the diagnosis of infectious diseases, identification of genetic mutations, drug discovery, forensics, and food technology [1–3]. For analyzing specific DNA sequences, reliable techniques based on either direct sequencing or DNA hybridization have been developed [2,3]. DNA sequencing is the process of determining the precise order of four nucleotides bases (adenine, guanine, cytosine, and thiamine) in a strand of DNA. DNA sequencing technology based on 2-D thin-layer chromatography was invented by Maxam and Gilbert and Sanger *et al.* in the 1970s [4,5]. The Maxam–Gilbert sequencing method rapidly became popular owing to the short sequence-reading times involved and because purified DNA could be directly used in this method. However, the requirement of using large amounts of purified DNA and complicated purification steps, combined with a shortage of available sequencers, limited the use of this method. Other major challenges associated with the Maxam–Gilbert method have been the extensive use of hazardous chemicals and difficulties with sample scale-up. By contrast, the chain-termination method developed by Sanger and coworkers made DNA sequencing comparatively more practical, because it required lesser amounts of purified DNA than the Maxam–Gilbert method did and it also provided multiple options for labeling the sequencing template. Of the two methods, the Sanger method is more efficient, uses fewer toxic chemicals, and requires the use of lower amounts of radioactivity. Furthermore, radioactive phosphorus labeling or the use of a primer labeled at the 5′ end with a fluorescent dye allows an optical set-up to be employed in the sequencing performed using the Sanger method; this facilitates easy analysis and the use of inexpensive automation. In order to enhance the sensitivity of this method, dye-terminator sequencing chemistry has been introduced [6–8]. However, dye-terminator sequencing has limited practical utility owing to the “dye effect” that arises from the difference in the incorporation of the dye-labeled chain terminators into the DNA fragment, which generates unequal peak heights and shapes in the DNA sequencing chromatogram. DNA sequencing by hybridization onto a solid support (e.g., nitrocellulose, nylon membrane, or lysine-coated glass slide) performed using fluorescently or radioactively tagged DNA became a common method for DNA analysis in the early 1990s [9,10]. This detection method appeared to be a promising tool for the real-time analysis of multiple DNA sequences, and it depended on the anchoring of multiple DNA-specific probes onto solid surfaces [11–13]. Such an array system might be useful in genome-wide genetic mapping, physical mapping, proteomics, and gene expression studies. However, the main challenges involved in using solid supports are the lack of commonly used DNA probes in “user-friendly” assays and an immobilization method that is fully compatible with the hybridization process, and low sensitivity and reproducibility [14]. To enable rapid, sensitive, and label-free DNA detection, numerous approaches have been suggested based on optical [15–17], acoustic [18], and electrochemical techniques [19–21].

Electrochemical methods are typically inexpensive and rapid methods that allow distinct analytes to be detected in a highly sensitive and selective manner [22–25]. Although electrochemical DNA sensors exploit a range of distinct chemistries, they all take advantage of the nanoscale interactions among the target present in solution, the recognition layer, and the solid electrode surface. This has led to the development of simple signal transducers for the electrochemical detection of DNA hybridization by using an inexpensive analyzer. DNA hybridization can be detected electrochemically by using various strategies that exploit the electrochemistry of the redox reaction of reporters [26] and enzymes immobilized onto an electrode surface [27], direct or catalytic oxidation of DNA bases [28–31], electrochemistry of nanoparticles [32–35], conducting polymers (CPs) [35–37], and quantum dots [38].

CPs are organic conjugated polymers that feature an extended π-orbital system through which electrons can move from one end of the polymer to the other. In 2000, H. Shirakawa, A. MacDiarmid, and A. Heeger were awarded the Nobel Prize in chemistry for their revolutionary research on the conductive behavior of polymers and provocative research based on CPs. Unlike saturated polymers, CPs exhibit several distinctive properties such as excellent electrical conductivity, low ionization potentials, and high electron affinity. The electrical conductivity of CPs is responsible for the excitation of polarons, bipolarons, and solitons during the doping processes. The ground state p-bonds (*p* − p*) of CPs are partially localized as a result of Peierls distortion [39]. However, depending on the doping concentration, the formation of polarons, bipolarons, and solitons creates distinct band gaps between the self-localized excitations and the localized electronic sates. CPs also exhibit very high flexibility, which can be modulated together with their electrical properties by using appropriate chemical modeling and synthesis [40–42]. These distinctive properties of CPs have broadened their application in various technological fields, such as in the design of light-emitting diodes [43], anti-static coating [44], electrochromic devices [45], solar cells [46], anti-corrosion coatings [47], chemical sensors and biosensors [48], and drug-release systems [49,50]. To date, diverse CPs have been developed and used in sensing applications, such as poly(acetylene), polypyrrole (PPy), polythiophene (PTh), poly(terthiophene), polyaniline (PANI), poly(fluorine), poly(3-alkylthiophene), poly(tetrathiafulvalene), poly(naphthalene), poly(p-phenylene sulfide), poly(para-phenylene vinylene), and poly(thionine) (PTH); these CPs are reviewed elsewhere [51]. Previous studies have also examined the growth and stabilities of PANI [52,53], PPy [54,55], poly(azulene) [56], and PTh [57,58]. Apart from these commonly used CPs, various reversibly doped and undoped CPs, which exhibit considerable changes in conductivity, have been studied using electrochemical methods [59]. The electrical conductivity of CPs depends substantially on the pH and the applied potentials, which can vary over several orders of magnitude [60]. Moreover, the grafting of organometallic compounds can aid in the tuning of the physical properties of CPs [61–64]. The electronic structure, chain conformation, and orientation of CPs can cause extremely sensitive changes in the polymeric chain environment of CPs. For example, a change in the delocalized electronic structure of CPs during DNA hybridization alters their optical and electrical properties [65]. These advantages offered by CPs make them suitable for developing sensitive electrochemical DNA hybridization sensors. In this article, we comprehensively review recent works on various CPs, as well as their application and implementation for electrochemical DNA sensing. Furthermore, we discuss the most commonly used methods of immobilizing DNA probes for developing DNA biosensors along with the transduction mechanism employed.

## 2. Immobilization Techniques Used for Developing DNA Biosensors

**Figure 1.**
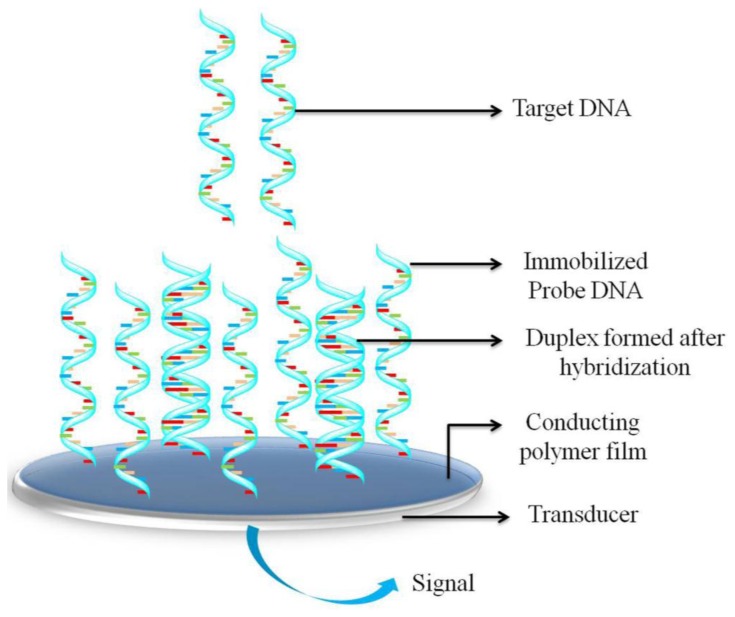
Schematic representation of a general electrochemical DNA sensor based on conducting polymers.

To enhance the sensitivity of DNA biosensors, the DNA probe used must be sufficiently immobilized [66]. Nonspecific adsorption and stabilization of the immobilized DNA probe are crucial for achieving high sensitivity and specificity. Moreover, minimizing nonspecific adsorption is essential for ensuring the high reactivity, accessibility, orientation, and stability of surface-confined DNA probes [66]. DNA probes immobilized on sensor surfaces can be used in a manner similar to enzyme-based biosensors; these probes are immobilized by means of adsorption, covalent immobilization, or avidin (or streptavidin)-biotin interaction [67,68]. Figure 1 shows the unique design of a CP-based DNA sensor. In this sensor, single-stranded DNA (ss-DNA) probes are immobilized on or within a CP layer, and target DNA base-pairing to the probe generates a recognition signal that can be recorded using an electrode (e.g., an electrode made of gold, platinum, or glassy carbon). Identification occurs at the CP/electrolyte edge, and the generated recognition signal reaches the transducer through the CP layer. In this section, we discuss the various methods used for immobilizing a DNA probe in order to develop a DNA hybridization sensor.

### 2.1. Adsorption

Adsorption is the simplest immobilization method in which a DNA probe can be immobilized without any modification of the probe [69]. Hirayama *et al.* developed an enhanced and simplified dry-adsorption protocol for DNA probe immobilization that increased hybridization sensitivity [70]. The efficiencies of DNA adsorption and retention were increased 1.4–6.5 and 4.2–19.6 times, respectively, compared with the efficiencies achieved using conventional methods such as incubation and decantation. Moreover, the use of this simple protocol reduces the consumption of DNA and increases the hybridization efficiency substantially. Another method involves the use of the amino group of the natural cationic chitosan polymer that can readily form a strong complex with the negatively charged phosphate backbone of DNA [71]. Xu *et al.* successfully immobilized a DNA probe labeled with aminoferrocene (AFC) on a chitosan-modified glassy carbon electrode (GCE) by means of adsorption [72]. The AFC-labeled DNA probe formed a duplex only with the complementary target DNA, and the detection limit was 2.0 nM. DNA that is either physically or chemically adsorbed onto a solid electrode surface can also be used for studying the electrochemical behavior of DNA and its interaction with other molecules [73–76]. For example, Azek *et al.* developed a disposable DNA sensor by physically adsorbing amplified human cytomegalovirus DNA onto a screen-printed electrode (SPE) [77]. The extent of hybridization of the target DNA was determined using horseradish peroxidase-conjugated streptavidin, and the detection limit was measured to be 6 × 10^−7^ nM.

DNA probes and DNA composites can also be immobilized on electrodes by applying an electric potential [78,79]. Wang *et al.* electrochemically adsorbed a DNA probe on an electrochemically pretreated carbon-paste electrode (CPE) at an applied potential of −0.5 V (*vs.* Ag/AgCl) [80]. This DNA sensor requires only nanogram quantities of DNA owing to the low background response of the potentiometric-stripping mode. Recently, Wu *et al.* electrodeposited silver nanoparticle-DNA composites at a controlled dimension on a GCE by reducing silver in the presence of DNA [81]. The inclusion of DNA with the silver nanoparticles prevents nanoparticle aggregation and enhances the catalytic activity. Lahiji *et al.* electrochemically deposited a uniform DNA-carbon nanotube (CNT) composite on an Au substrate by maintaining a +0.5 V potential [82]. This immobilization technique does not require prior DNA or substrate functionalization, and this is combined with a new method of generating a modified DNA electrode that offers the advantage of the high electron-transporting capacity of CNTs for sensing.

### 2.2. Covalent Immobilization

DNA probes have frequently been immobilized through covalent binding to various solids [83–88]. Ligaj *et al.* developed a stearic acid-modified conventional CPE in order to covalently immobilize DNA probes through an ethylenediamine linker [86]. This process does not alter the structural flexibility of the DNA probe, and it enhances DNA hybridization. Raymond *et al.* developed a simple and specific method that does not require the labeling of the target before hybridization [87]. The amino-linker of the probe DNA allows it to be covalently attached to a functionalized glass surface. Functionalized CP films, such as poly(3-pyrrolylacrylic acid) (Py-*co*-PAA), poly(5-(3-pyrrolyl) 2,4-pentadienoic acid) (Py-*co*-PPDA), and poly(3-pyrrolylpentanoic acid) (PPA), were previously used for covalently immobilizing a DNA probe [88]. Based on the principle of chemisorption, thiol-metal interactions have also been frequently employed to covalently bind thiol-functionalized DNA probes onto gold surfaces [89–92].

The distinctive electrical, thermal, chemical, mechanical, and 3-D spatial characteristics of CNTs suggest that it is possible to construct DNA biosensors with high sensitivity, selectivity, and multiplexing by exploiting Watson–Crick base-pairing [93]. Rodriguez *et al.* covalently attached single-walled CNTs (SWNTs) to a gold surface modified with 11-amino-1-undecanethiol (AUT) and subsequently immobilized a DNA probe on the Au/AUT/SWNTs through covalent linkage [94]. The interaction and covalent immobilization of DNA probes on functionalized CNTs and CNT/CP-composite-modified electrodes have been widely studied [95,96]. Yang *et al.* developed a sensitive electrochemical DNA sensor based on the synergistic effects of a PANI nanofiber and a multiwall CNT (MWNT) composite on a chitosan film [97]. The covalent immobilization of the DNA probe on the PANI-MWNT-composite film enhanced DNA hybridization, which was highly reproducible and stable.

### 2.3. Avidin/Streptavidin-Biotin Interaction

**Figure 2.**
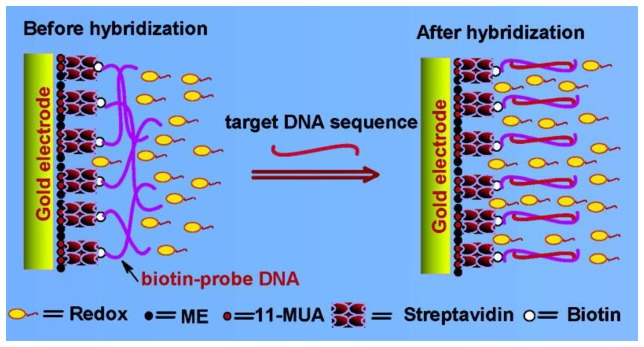
Schematic illustration of DNA probe immobilization through avidin-biotin chemistry and the hybridization of target DNA on a self-assembled monolayer (SAM)-modified Au electrode. (Reproduced with permission from [99]. Copyright 2014, American Chemical Society.)

Avidin and streptavidin are large tetrameric proteins containing four identical biotin-binding sites that can be used for forming tetravalent avidin/streptavidin-biotin bonds in order to develop DNA-coated electrodes; in these electrodes, surface-confined avidin/streptavidin reacts with biotinylated DNA [98]. Because of the innate aqueous immobility of the avidin-biotin complex, this system is easy to use. Pan *et al.* generated a mixture of self-assembled monolayers (SAMs) by using 2-mercaptoethanol (ME) and 11-mercaptoundecanoic acid (MUA) on an Au electrode and attached a DNA probe to the activated MUA through streptavidin-biotin chemistry, as shown in Figure 2 [99]. Caruso *et al.* employed the quartz crystal microbalance (QCM) technique in order to immobilize biotin-DNA on an avidin-modified QCM electrode [100]. Several strategies have been developed for attaching biotin to modified CP electrodes, including the biotin-sandwich technique for immobilizing DNA [101–106]. Guillerez *et al.* designed a DNA sensor by using an electropolymerized biotinylated-PPy film [101]. Biotinylated DNA probes were immobilized onto PPy-biotin films through an intercalated layer of avidin (PPy-biotin/avidin/DNA probe). Furthermore, to detect DNA hybridization, fluorescently labeled or non-labeled avidin and biotinylated DNA probes have also been immobilized on a biotin derivative (photobiotin)-modified poly(dimethylsiloxane) (PDMS) chip by using biotin/avidin/biotin chemistry [106].

## 3. Transduction Methods

Sensing and/or transduction must occur in order to convert a recognition event into a readable signal. Depending on the type of measurement, transduction can occur by electrochemical, optical, mass-based, or thermal means [107–109]. Among these, electrochemical transduction has been shown to be appropriate for DNA sensing, wherein a biorecognition event directly gives rise to an electrical signal, and this allows the sensing system to be miniaturized [110]. Recently, various convenient systems for analysis and on-site monitoring have been developed using distinct solid contact materials such as gold, ferrocene-labeled PNAs (peptide nucleic acids), CNTs, and CPs [111–113]. CPs have been widely used because they can create a high redox capacitance that makes the recorded signal highly stable [114–116]. The transduction mechanism of poly(3,4-ethylenedioxythiophene) (PEDOT) was comprehensively studied using various electrochemical techniques, including electrochemical impedance spectroscopy (EIS) [117,118]. Although CPs clearly offer advantages over other transducing materials, a few drawbacks are also associated with CPs depending on their type. For example, PPy exhibits slight chemical instability in the presence of certain components of ambient media, such as oxygen, acids, bases, redox reactants, nonreactive ions, and surfactants [119,120]. The possible formation of a water layer at the boundary between CPs and polymeric ion-selective membranes is also a hurdle that must be addressed [121]. In the case of CP-based electrochemical DNA sensors, the polymers not only serve as an immobilization template, but also actively participate in signal transduction. When using electrochemical methodologies, reversible doping and dedoping of CPs markedly alter the electrochemical responses. Doping and dedoping modulate the interaction of the probe-target complex and can regulate the sensitivity and stability of DNA detection [122,123]. The change in the signal after probe-DNA immobilization and target-DNA hybridization can also be quantified using diverse approaches, such as by measuring the change in current as a function of the applied potential (voltammetry), the change in current at a fixed applied potential (amperometry), or the change in conductivity (conductometry), impedance (impedimetry), or potential (potentiometry). Among these transduction methods, voltammetric methods (e.g., cyclic voltammetry (CV), differential pulse voltammetry (DPV), and square wave voltammetry (SWV)) are most widely used for the detection of DNA hybridization. Voltammetric measurement requires the use of two- or three-electrode electrochemical cell systems together with a potentiostat, which allows the application of the potential and the measurement of the resultant current. Voltammetry such as CV, DPV, and SWV depend on the pattern of the applied potential, which also potently controls the sensitivity of the current response.

## 4. Development of DNA Sensors Based on Distinct CPs

### 4.1. DNA Sensors Based on Polypyrrole and Its Derivatives

PPy is formed from a number of connected pyrrole ring systems and is highly biocompatible. PPy synthesized at neutral pH is extensively used as a versatile immobilization matrix in the design of biosensors, such as catalytic biosensors, immunosensors, and DNA sensors and in molecular-imprinting technologies [124]. When deposited on the electrode surface, PPy provides an effective DNA-sensing platform, in which PPy itself acts as an interface for the attachment of the DNA probe. Notably, PPy-modified electrodes have also facilitated the development of indicator- and label-free detection of DNA [125]. Livache *et al.* developed a novel method for electro-synthesizing a PPy-DNA composite through co-polymerization [126]. To synthesize a PPy film harboring covalently linked DNA, a mixture of pyrrole and a pyrrole bearing a specific DNA probe was electrooxidized. This was the first study to detect DNA hybridization on the surface of modified macroelectrodes. Soon after this study, the same group also generated a DNA chip constructed of three components: silicon chips bearing a matrix of 50-μm or 4-μm microelectrodes for genotyping hepatitis C virus (HCV), a QCM, and a non-patterned gold/glass slide featuring 500-μm spots [127]. Wang *et al.* described the incorporation of DNA dopants into a PPy network by using an electrochemical QCM (EQCM) and showed that it exhibited a strong affinity for target DNA [128,129]. Youssoufi *et al.* developed a new type of electrochemical DNA hybridization sensor based on DNA-functionalized PPy [130]. The prepared PPy precursor contained a loosely bound ester group that was directly substituted with an amino-labeled DNA probe of various sequence lengths. The electrochemical response of this sensor was analyzed in aqueous media containing distinct target DNA sequences. The voltammetric signals obtained for DNA-PPy remained unchanged in the presence of a noncomplementary target DNA sequence; however, the signal changed considerably when a complementary target DNA was added. This was quantified using amperometry, and the detection limit of the biosensor was determined to be approximately 1 × 10^−2^ nmol in the absence of any signal processing. Other researchers also developed a similar type of DNA hybridization sensor by functionalizing PPy; these researchers introduced PPy nanotubes in which PPy was functionalized with, for example, poly[pyrrole-*co*-4-(3-pyrrolyl) butanoic acid] or carboxylic acid [131–133]. The acid-functionalized PPy is a favorable alternative for fabricating label-free sensors because it enables versatile immobilization of DNA, proteins, and enzymes by using various pendant groups: −SH, −NH_2_, and −COOH. Peng *et al.* prepared a poly[pyrrole-*co*-4-(3-pyrrolyl) butanoic acid]-modified platinum electrode for DNA hybridization, which exhibited high electroactivity in aqueous medium [131]. An NH_2_-substituted DNA probe was covalently grafted onto the surfaces of this polymer in a one-step procedure. Komarova *et al.* developed a prototype amperometric sensor for the detection of a biowarfare pathogen, the virus *Variola major*, based on DNA-doped ultrathin PPy films [134]; the investigators determined that thinner films harboring smaller or more highly concentrated dopant ions produced stronger amperometric signals than did thicker films bearing larger or less concentrated dopant ions. After the film surface was blocked with fragmented calf-thymus DNA, the nonspecific signal disappeared completely when ultrathin (Langmuir–Blodgett) films were tested; however, the specific signal from the complementary DNA remained unaffected. Under optimal conditions, the detection limit for the target DNA was 16 × 10^−3^ nM. Ease of use and rapid detection are the primary advantages of this sensor; however, steric hindrance and poor accessibility of the probe to the analyte in the film can reduce hybridization efficiency and substantially limit sensitivity and selectivity [68].

Several groups have developed DNA sensors based on PPy and PPy derivatives by using a modified fluorine-doped tin oxide (FTO) electrode [135–137]. Eguiluz *et al.* developed a PPy/FTO electrode and used Ag/Au-nanoparticle labels to detect *Alicyclobacillus acidoterrestris* in pure cultures by means of reverse-transcription polymerase chain reaction (RT-PCR) [136]. The sensor sensitivity could also be enhanced by performing asymmetric nested RT-PCR of the amplicon and using Ag/Au-based electrochemical detection, which was able to detect 2 colony-forming units/mL of spores. In this electrochemical bioassay, the detection and quantification limits for the target *A. acidoterrestris* were 7.07 and 23.6 nM, respectively. Riccardi *et al.* developed a new type of label-free PPy-based DNA sensor for identifying HCV [137]; in this approach, HCV is detected through the electrostatic modulation of the ion-exchange kinetics of PPy films. Here, the PPy layer was electropolymerized in order to immobilize a synthetic, single-stranded, 18-mer HCV genotype-1-specific probe DNA on a 2,5-bis (2-thienyl)-*N*-(3-phosphoryl-*n*-alkyl)pyrrole film. HCV DNA sequences (244-mer) obtained through RT-PCR amplification of the original viral RNA were examined based on the disruption of the ion-exchange properties of the PPy film. However, with this sensor, the selectivity, sensitivity, and reproducibility of DNA detection were poor.

In order to overcome the poor selectivity, sensitivity, and detection limit of DNA sensors, researchers have introduced PPy composites, such as PPy-CNTs, PPy-nanoparticles, PPy-nanoengineered materials, and pyrrole-derivative bilayers [138–140]. Xu *et al.* developed an impedimetric DNA biosensor based on a GCE modified with a PPy-MWNT composite [138]. COOH-MWNTs and PPy were electrodeposited on the GCE to facilitate the immobilization of the NH_2_-DNA probe. The hybridization reaction of this DNA/PPy/MWNT-COOH/GCE results in a decrease in impedance, which is attributed to the electron-transfer resistance through double-stranded DNA (ds-DNA) being lower than that through ss-DNA. The PPy/MWNT-COOH-modified electrode exhibited high electron-transport capacity and also featured an increased specific surface area. Consequently, the sensitivity and selectivity of DNA hybridization were increased and the detection limit was 5.0 × 10^−3^ nM. Table 1 summarizes the characteristics of some of the reported electrochemical DNA sensors based on PPy and PPy derivatives, together with their immobilization methods, detection method, detection limit, and sensitivity.

### 4.2. DNA Sensors Based on Polythiophene and Its Derivatives

**Figure 3.**
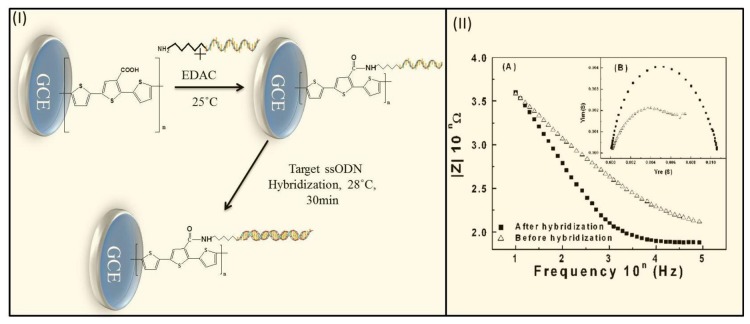
Schematic representation of the immobilization of probe DNA and the hybridization of a target sequence (**I**), and plots of (A) impedance and (B) admittance before and after hybridization in a phosphate buffer solution (**II**). (Redrawn and reproduced with permission from [146]. Copyright 2014, American Chemical Society.)

PTh and functionalized PTh demonstrate a variety of remarkable solid-state properties and hold tremendous potential for use in molecular electronic devices, solid-state batteries, and sensors [144,145]. Carboxylic acid- and ester group-functionalized PTh polymers (e.g., 3′-carboxyl-5,2′,5′,2″-terthiophene, poly(thiophen-3-yl-acetic acid 1,3-dioxo-1,3-dihydro-isoindol-2-yl ester) (PTAE), and 3-((2′:2″,5 ″:2‴-terthiophene)-3″-yl) acrylic acid (TAA)) have been widely used for developing electrochemical DNA sensors [146–149]. Lee *et al.* used a poly(3′-carboxyl-5,2′,5′,2″-terthiophene)-modified GCE and reported that only a short hybridization time (1 h) was required [146]. The amine group linked to the 5′ end of the DNA probe (a 19-mer) was covalently attached with the carboxyl (-COOH) group-terminated polymer, which corroborated the hybridization of the target DNA (Figure 3I). The hybridization of fully complementary target DNA induced a significant decrease of the impedance values (Figure 3II). The difference in impedance values before and after hybridization of target DNA can be ascribed by the change in conductivity and capacitive current. This method is more advantageous than other methods because of its selectivity, short response time, and minimal use of intercalators and fluorescent tags. Cha *et al.* described a synthetic route for thiophen-3-yl-acetic acid 1,3-dioxo-1,3-dihydro-isoindol-2-yl ester (TAE), which can be readily electropolymerized on a Pt chip electrode and allow for the direct substitution of its exiting group with a prosthetic group that contains a terminal amino group on the DNA probe [149]. The sensitivity of this sensor was 0.62 μA/nmol and the detection limit was 1 nmol.

Peng *et al.* fabricated a poly(TAA)-modified DNA sensor that exhibited a qualitatively unique response when compared to functionalized PPy sensors [148,149]. The applicability of these two polymers as active substrates for DNA sensors was confirmed by covalently attaching NH_2_-DNA probes to the −COOH group of both polymers. Here, the hybridization of complementary DNA can be detected by an increase in the admittance without the requirement for an indicator or any sample modification. Experimental results suggested that PPy functionalized with long unsaturated carbon side chains exhibited more favorable DNA-sensing properties and a larger difference in the impedance signal. This difference was due to the disparities in the movement of the dominant ions (CF_3_SO_3_^−^ and ClO_4_^−^) at the interface of the polymer film and the electrolyte, which was confirmed using an EQCM.

**Figure 4.**
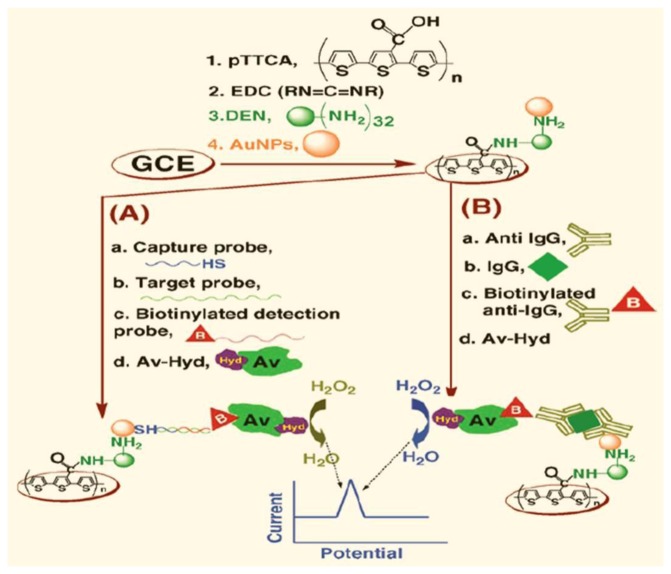
Schematic illustration of the poly-5,2′:5′,2″-terthiophene-3′-carboxylic acid (pTTCA)/ dendrimer (DEN)/ Au nanoparticles (AuNP)/biomolecule-linked avidin-hydrazine assembly developed for (**A**) DNA and (**B**) protein sensors, based on the electrocatalytic activity of hydrazine. (Reproduced with permission from [154]. Copyright 2014, American Chemical Society.)

Considerable emphasis has been placed on producing portable and inexpensive devices for DNA detection because of their importance in forensics, medical diagnostics, and evolutionary studies [150–153]. Shiddiky *et al.* developed an ultrasensitive technique for detecting DNA and proteins based on poly-5,2′:5′,2″-terthiophene-3′-carboxylic acid (pTTCA) (Figure 4) [154]. Dendrimer (DEN) and hydrazine were covalently linked to the pTTCA film and the signal was amplified by the pTTCA/DEN assembly loaded with Au nanoparticles (AuNPs). The target DNA- or protein-linked hydrazine labels (avidin-hydrazine) adsorbed onto the pTTCA/DEN film, and DPV measurements revealed a linear dynamic range for the electrocatalytic detection of DNA and protein. The simplicity, low detection limit, and reproducibility (RSD < 4.3% for *n* = 10) of the sensor make this a promising tool that can be developed in the future for practical applications.

Functionalized PTh was also used for developing a DNA sensor. Fang *et al.* developed a novel methodology for detecting DNA by using ferrocene-functionalized PTh deposited on a nanogold-modified electrode [155]. Nanogold-modified electrodes substantially increase the quantity of immobilized PNA probes and thus cause an increase in the electrical signal. Positively charged ferrocene-functionalized PTh does not bind electrostatically with the PNA probes because of the absence of anionic phosphate groups. This limitation can be resolved by performing an initial DNA–PNA hybridization. Adsorption of cationic PTh onto the DNA backbone results in the generation of a detectable hybridization-recognition signal in DPV. Thus, PNA could be used as a highly sensitive, selective, and reversible coupling substrate for DNA immobilization. Table 2 summarizes the characteristics of electrochemical DNA sensors based on PTh and PTh derivatives, together with their immobilization techniques, detection method, detection limit, and sensitivity.

### 4.3. DNA Sensors Based on Polyaniline and Its Derivatives

The environmental stability of PANI and its easy synthesis (either chemically or electrochemically) have broadened the application of PANI as a chemical sensor [157,158] and biosensor [159–161]. PANI is the best-recognized semi-flexible rod-like CP with chemically and structurally flexible –NH_2_ linkage on its surrounding molecules, thus making it suitable for binding biomolecules [159]. PANI undergoes two redox reactions and can be functionalized, and this makes it a favorable material for DNA sensing. The electrical conductivity of PANI has been established to strongly depend on pH, and most previous studies on PANI were performed at a pH below 4.0. However, using a neutral pH solution is critical for developing biosensors because most biocatalytic and immunological reactions occur at neutral pH [162]. Therefore, a challenge is to incorporate biological molecules in the conventional pH-dependent PANI. Research has shown that N-substituted aniline does not exhibit pH sensitivity because an alkyl chain is covalently bound to the nitrogen atom in order to prevent the formation of an emeraldine base (the deprotonated form). Moreover, self-doped PANI, commonly referred to as sulfonated PANI, exhibits redox activity even at neutral pH [163].

Wu *et al.* synthesized PANI-intercalated graphite-oxide nanocomposites (PANI/GO) enveloped in CPE [164]. This PANI/GO-modified CPE displayed electrochemical activity and two sharp peaks at 668 and 207 mV in SWV measurements. These results indicated that ss- and ds-DNA transformed the redox characteristics of PANI/GO, and this could be used to monitor probe immobilization and the hybridization of complementary DNA, with the hybridization peak occurring at −270 mV. DNA detection performed using the PANI/GO-modified CPE was highly stability and reproducible. Gu *et al.* developed an impedimetric DNA hybridization sensor based on a PANI/polyacrylate (PANI/PAA)-modified boron-doped diamond (BDD) electrode [165]. An ultrathin film of the PANI-PAA copolymer was electropolymerized onto BDD surfaces in order to enhance the availability of the −COOH for binding the DNA probe. The hybridization event was sensed based on the direct oxidation of guanine and adenine in the DNA double helix.

Immobilization of probe DNA on a polymer matrix revealed the limitations in the selectivity and specificity of hybridization [166]. In this context, PNA is recognized to provide enhanced stability and specificity in the detection of targets containing a single mismatch [167]. Gao *et al.* developed a novel signal-amplification method for ultrasensitive detection of DNA, which involved enzymatically catalyzed PANI formation and template-guided deposition for enhancing DNA hybridization [168]. The hybridization was quantified by examining the electroactivity of the deposited PANI by using SWV. This DNA sensor was extremely sensitive—it had a femtomolar detection limit—and it was highly selective for sequences mismatched by one, two, and three bases. The biosensor was used for detecting His4, RCA1, and GAPDH, and the results obtained were similar to those obtained from northern-blotting analysis of the same samples.

**Figure 5.**
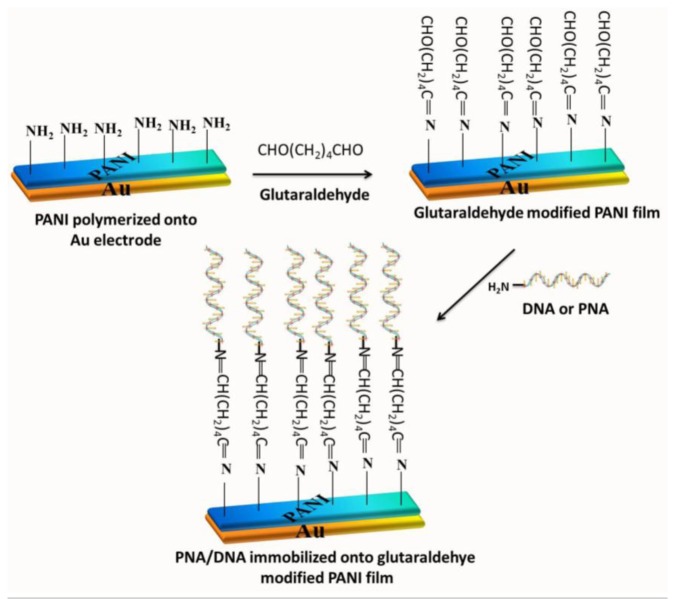
NH_2_-DNA and peptide nucleic acid (PNA) immobilization on polyaniline-coated gold film. (Redrawn with permission from [174]. Copyright 2014, American Chemical Society.)

An ultrasensitive technique for detecting DNA hybridization has also been developed by using PANI nanowires or nanotubes or methylene blue (MB) as an indicator [169,170]. MB was used to distinguish between ss- and ds-DNA by using various electrochemical techniques [171,172]. MB specifically binds to unpaired bases of DNA/PNA. The redox reaction of MB was used for monitoring the native and denatured states of DNA [173]. Prabhakar *et al.* covalently immobilized 20-base-long NH_2_-DNA and PNA probes on a PANI/Au electrode in order to detect DNA hybridization and used MB as an indicator (Figure 5) [174]. PNA-PANI/Au and DNA-PANI/Au electrodes were used for detecting the presence of complementary target *Mycobacterium tuberculosis* by using SWV, and the response time was short (30 s). The study revealed that the PNA electrode exhibits a higher affinity for complementary DNA sequence and an improved detection limit and higher specificity than do electrodes used in other methods. Table 3 lists the characteristics of electrochemical DNA sensors based on PANI and PANI derivatives, together with their immobilization techniques, detection method, detection limit, and sensitivity.

### 4.4. DNA Sensors Based on Quinone and Its Derivatives

Quinone is an electronically conductive redox polymer that has attracted substantial interest as a chemical sensor [177] and biosensor [178,179] because of its capacity to transport charges inside films and the nature of the ionic flux at the interface of the polymer and the solution (anionic and cationic, respectively). The characteristics of quinone-based polymers differ from those of conventional p-doped electronically conducting polymers (ECPs). The sensitivity of ECP-based biosensors depends on the amplification of the interaction between the electrochemical transducer and biomolecules [180].

**Figure 6.**
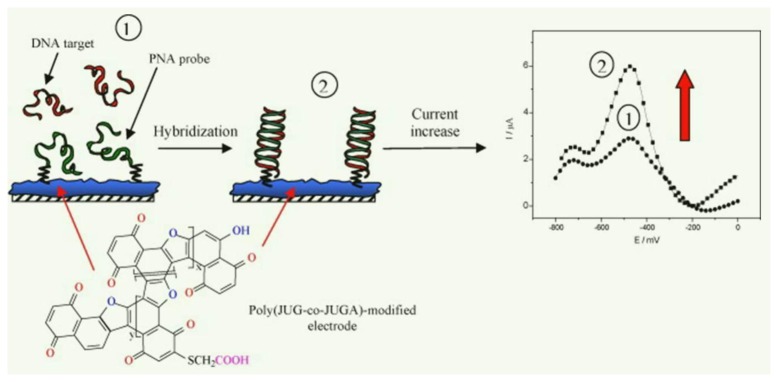
(**1**) Immobilization of the peptide nucleic acid (PNA) probe; and (**2**) hybridization of the target DNA onto poly(JUG-*co*-JUGA)-modified glassy carbon electrode (GCE) together with the corresponding square wave voltammetry (SWV) signals for (**1**) PNA probe immobilization, and (**2**) target DNA hybridization. (Reproduced with permission from [182]. Copyright 2014, Elsevier.)

Piro *et al.* constructed a new electroactive film, poly(JUG-*co*-JUGA), by co-electrooxidizing 5-hydroxy-1,4-naphthoquinone and 5-hydroxy-3-thioacetic acid-1,4-naphthoquinone [180]. This poly(JUG-*co*-JUGA) copolymer presents both electroactive and chemically reactive groups for sensing DNA and L-lactate [181]. An NH_2_-DNA probe was covalently immobilized on poly(JUG-*co*-JUGA) and the electroactivity of the quinone group was used for detecting hybridization. The main feature of this DNA hybridization sensor is the transformation between ss- and ds-DNA at the solution/polymer interface. Therefore, the rate of charge (ion) diffusion from the solution to the polymer/solution interface is primarily affected by hybridization. After hybridization, the current increases in SWV measurements because of electrostatic and/or steric effects. Piro and coworkers also examined PNA probe-based DNA hybridization sensors (Figure 6) [182]. The PNA probe was covalently attached to poly(JUG-*co*-JUGA), and upon hybridization with the target complementary DNA, the flexibility of the PNA probe was altered, which caused changes in the electrochemical signal at the polymer/solution interface. This simple and reagent-free DNA hybridization sensor can discriminate single mismatches and can be regenerated after a simple dehybridization step. The main drawback of this sensor is its detection limit, 10 nM, which does not constitute the theoretical limit. In order to improve the detection limit, Acevedo *et al.* fabricated a sequential multilayer CNT that featured an increased area for the oxidation of soluble redox couples [183]. The electropolymerization of quinine and quinone derivatives onto the MWNT-modified electrode produced an interpenetrated CP/CNT network that is electroactive in both aqueous and nonaqueous media. The effective current response was enhanced up to 19 times, which increased the sensitivity and lowered the detection limit.

### 4.5. DNA Sensors Based on Miscellaneous Conducting Polymers and Their Derivatives

**Figure 7.**
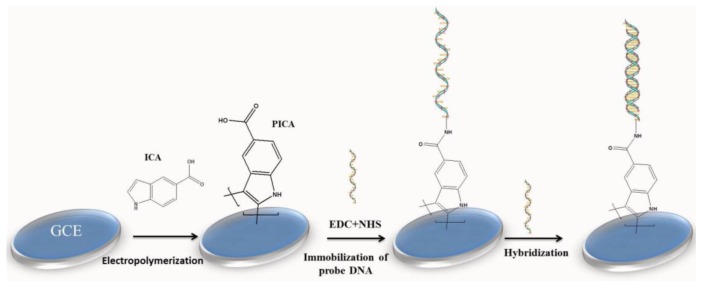
Schematic representation of the preparation of an electrochemical DNA sensor based on a poly(indole-5-carboxylic acid) (PICA) conducting polymer. (Redrawn from [187]. Copyright 2014, Elsevier.)

Poly(triamine) (PTyr) contains one primary aliphatic amine per triamine moiety, which represents an extremely high concentration of surface reactive sites for biomolecule immobilization [184]. Tran *et al.* first prepared a PTyr film by electrooxidizing 4-hydroxiphenylethylamine in perchloric acid, which left one reactive amine group per moiety [185]. DNA was immobilized on the polymer film through a phosphoramidate covalent bond [186], and this yielded a high surface concentration of DNA (∼500 pmol/cm^2^). DNA can also readily bind to PTyr through nonspecific adsorption, and thus differentiating between adsorbed and covalently bonded DNA could be challenging. However, DNA probes that were weakly adsorbed were removed by washing with SL salmon-testis DNA. The remaining covalently bonded DNA probes were used for the hybridization of GEM DNA, a complementary DNA sequence derived from HIV gag protein. For detecting DNA hybridization, Li *et al.* prepared a new type of conjugated CP, poly(indole-5-carboxylic acid) (PICA), on GCE by means of anodic oxidation (Figure 7) [187]. PICA exhibited optimal electrochemical behavior and thermal stability, with a conductivity of 10^−2^ S/cm and high redox activity compatible with the concept of molecular-wire transduction. The PICA-modified sensor showed comparable sensitivity and its detection limit was 1.0 nM, which can be further improved by increasing the side chain length because longer side chains permit greater freedom of movement and more enhanced hybridization [35].

Certain other CPs have been synthesized and employed in DNA sensing applications in which fluorescence techniques are used. These CPs include poly({2,5-bis[3-(N,N-diethylamino)-1-oxapropyl]-para-phenylenevinylene}-alt-para-phenylenevinylene)dibromide [188], poly(9,9-bis(6′-N,N,N-trimethylammonium)-hexyl)-fluorene phenylene) [189], and poly(fluorene-*co*-phenylene) [190]. The potential application of these CPs for the development of an electrochemical DNA sensor is in high demand.

## 5. Conclusions and Outlook

This review has summarized the diverse strategies used for developing DNA biosensors by using electroactive CPs. CPs have been extensively used for developing electrochemical label-free methods of DNA detection. This is because CPs exhibit highly favorable electrical conductivity or charge-transport properties. Over the last decade, researchers have developed numerous types of CPs and their derivatives for highly sensitive and selective electrochemical detection of DNA. Adsorption, covalent immobilization, and avidin-biotin interactions have been used for developing DNA biosensors by modifying the electrode surface with CPs and their derivatives. To develop CP-based DNA sensors, regeneration of a surface-immobilized probe and the reuse of DNA biosensors must be addressed. The key factors that must be considered for probe immobilization are the following: the immobilization chemistry must be stable, the probes must retain their functionality after attachment, and immobilized biomolecules must maintain proper orientation and configuration [191]. CP films must be deposited on inert substrates because hydrophobic interactions and the consequential electrochemical oxidation and reduction accompanied by the movement of ions in and out of the CP film can result in the delamination of CP films. With regard to the immobilization of DNA probes, most detection methods that are used for developing DNA biosensors and microarrays are open to criticism [192]. CP-based DNA sensors are expected to be highly sensitive, selective, and reproducible and to enable multi-analyte determination.

Although we have focused on CPs and various CP-CNT composites, other materials could also be used for developing electrochemical DNA hybridization sensors. For example, graphene, a new allotrope of carbon composed of *sp*^2^-hybridized carbon atoms arranged in a honeycomb lattice, is an ideal 2-D material for developing DNA sensors. Graphene can be readily functionalized and doped with various functional groups (e.g., −COOH, −OH) and atoms, which might facilitate the immobilization of DNA probes in a biocompatible manner.

## Figures and Tables

**Table 1.** DNA sensors based on Polypyrrole (PPy), PPy derivatives, and PPy composites and their performance in the detection of DNA hybridization.

**Matrix**	**Immobilization Method**	**Detection Method**	**Detection Limit**	**Sensitivity**	**Ref.**
Copolymerizations of 5′ pyrrole-labeled DNA and pyrrole	DNA entrapment	Fluorescence microscopy	-	>10^−11^ M	[127]
Copolymerizations of DNA probe within PPy	DNA entrapment	CV/amperometry	>6 μg	1.08 nA/μg	[129]
Poly[3-acetic acid pyrrole, 3-*N* hydroxyphthalimide pyrrole]]	Covalent	CV	1 × 10^−2^ nmol	-	[130]
Poly[pyrrole-*co*-4-(3-pyrrolyl) butanoic acid]	Covalent	CV/ EIS	-	10.5, 3.0 and 1.7 μA/cm^2^/nM of complementary DNA for 23, 57, and 114 nm film thicknesses, respectively.	[131]
Carboxylic acid-functionalized PPy nanotubes (CPPy NTs)	Covalent	Photoluminescence	-	High sensitivity (ΔR/R_0_ = 1.7) even at low concentration (1 nmol) of target DNA	[132]
Poly [3-acetic acid pyrrole, 3-N-hydroxyphthalimide pyrrole]	Covalent	EIS	1 × 10^−3^ nmol	21.6 Ω cm^−2^/μM	[133]
PPy doped with an DNA	DNA entrapment	Chronoamperometry	16 × 10^−3^ nM	-	[134]
poly(Py-*co*-PAA)	Covalent	QCM/EIS	0.98 nM	-	[135]
PPy-DNA	DNA entrapment	CV/Linear Sweep Voltammetry (LSV)	-	-	[136]
2,5-bis(2-thienyl)-*N*-(3-phosphorylpropyl)pyrrole	DNA entrapment, Mg^2+^ ion serve as a linker	CV/LSV	1.82 × 10^−12^ nM	-	[137]
PPy/MWNTs	Carbodiimide cross linking between amine and carboxyl group	CV/EIS	5.0 × 10^−3^ nM	-	[138]
PPy–polyaniline–Au	HS-DNA bind on Au via Au-thiol chemistry	EIS	1.0 × 10^−4^ nM	-	[141]
PPy–poly(3,4-ethylenedioxythiophene)–Ag	HS-DNA bind on Ag via Ag-thiol chemistry	EIS	5.4 × 10^−6^ nM	-	[142]
Copolymer of PPy and 3-pyrrolylacrylic acid (PAA)	Covalent	EIS	-	-	[143]

**Table 2.** Polythiophene (PTh)- and PTh-derivative-based DNA sensors and their performance in the detection of DNA hybridization.

**Matrix**	**Immobilization Method**	**Detection Method**	**Detection Limit**	**Sensitivity**	**Ref.**
PTh	Covalent	Fluorescence	-	-	[144]
Poly(3′-carboxyl-5,2′,5′,2″-terthiophene)	Covalent	EIS	-	5.608 (ng/cm^2^)/Hz	[146]
Poly(thiophen-3-yl-acetic acid 1,3-dioxo-1,3-dihydro-isoindol-2-yl ester	Covalent	CV	1 nmol	0.62 μA/nmol	[149]
Poly (3-[(S)-5-amino-5-carboxyl-3-oxapentyl]-2,5-thiophenylene hydrochloride)	Hydrogen bond	Fluorometric	1 × 10^−2^ nmol	-	[151]
Cationinc PTh	Covalent	Fluorometric	3.6 × 10^−13^ nM	-	[152]
Poly(5,2′:5,2″-terthiophene-3′-carboxylic Acid)	Covalent	Electrophoresis	1.14 × 10^−4^ nM	0.20 nA (fg/μL)^−1^	[153]
PTh functionalized- methylene blue	HS-DNA bind on Au via Au-thiol chemistry	DPV	-	-	[156]

**Table 3.** Polyaniline (PANI)- and PANI-derivative-based DNA sensors and their performance in the detection of DNA hybridization.

**Matrix**	**Immobilization Method**	**Detection Method**	**Detection Limit**	**Sensitivity**	**Ref.**
PANI-intercalated graphite oxide nanocomposite	Covalent	SWV	-	0.77 μA/μg/mL	[164]
PANI/PAA-modified borondoped diamond (BDD)	Covalent	EIS	20 nM	-	[165]
Avidin modified-PANI	Avidin interaction	DPV	5 × 10^−10^ nmole	-	[166]
Polyaniline nanowire	Covalent immobilization	DPV	1 × 10^−3^ nM	-	[169]
PANI nanotube	Covalent	CV/DPV	1 × 10^−6^ nM	1 pM	[170]
Graphene/PANI	Non-covalent binding	DPV	3.2 × 10^−5^ nM	-	[175]
PANI-Au	HS-DNA bind on Au via Au-thiol chemistry	DPV	0.1 nM	-	[176]
